# Next-Generation Sequencing with Liquid Biopsies from Treatment-Naïve Non-Small Cell Lung Carcinoma Patients

**DOI:** 10.3390/cancers13092049

**Published:** 2021-04-23

**Authors:** Paul Hofman

**Affiliations:** 1 Laboratory of Clinical and Experimental Pathology, Université Côte d’Azur, CHU Nice, FHU OncoAge, Pasteur Hospital, 30 avenue de la voie romaine, BP69, CEDEX 01, 06001 Nice, France; hofman.p@chu-nice.fr; Tel.: +33-4-92-03-88-55 or +33-4-92-03-87-49; Fax: +33-4-92-88-50; 2 Hospital-Integrated Biobank BB-0033-00025, Université Côte d’Azur, CHU Nice, FHU OncoAge, 06001 Nice, France

**Keywords:** liquid biopsy, lung cancer, next-generation sequencing, targeted therapy

## Abstract

**Simple Summary:**

Screening for genomic alterations in treatment-naïve non-small cell lung carcinoma (NSCLC) is mainly done by tissue biopsy (TB), an invasive approach. However, it may not be possible to obtain a TB, the patient does not consent to it and/or the extracted nucleic acids are of poor quantity and/or quality for further genomic analyses, so a liquid biopsy (LB) is the only option to detect molecular target(s) for first-line treatment in these patients. However, a LB at diagnosis is still not often used in clinical centers since a TB is currently the gold standard approach for histological diagnosis, assessment of the PD-L1 status on tumor cells and evaluation of the molecular alterations. A number of different approaches are already available for the assessment of genetic abnormalities with LB, but next-generation sequencing (NGS) is the most promising. This review provides an overview of the main studies currently using LB NGS at diagnosis for NSCLC. We discuss its advantages and limitations in comparison with a TB and the perspectives for the future.

**Abstract:**

Recently, the liquid biopsy (LB), a non-invasive and easy to repeat approach, has started to compete with the tissue biopsy (TB) for detection of targets for administration of therapeutic strategies for patients with advanced stages of lung cancer at tumor progression. A LB at diagnosis of late stage non-small cell lung carcinoma (NSCLC) is also being performed. It may be asked if a LB can be complementary (according to the clinical presentation or systematics) or even an alternative to a TB for treatment-naïve advanced NSCLC patients. Nucleic acid analysis with a TB by next-generation sequencing (NGS) is gradually replacing targeted sequencing methods for assessment of genomic alterations in lung cancer patients with tumor progression, but also at baseline. However, LB is still not often used in daily practice for NGS. This review addresses different aspects relating to the use of LB for NGS at diagnosis in advanced NSCLC, including its advantages and limitations.

## 1. Introduction

**Figure 1:**
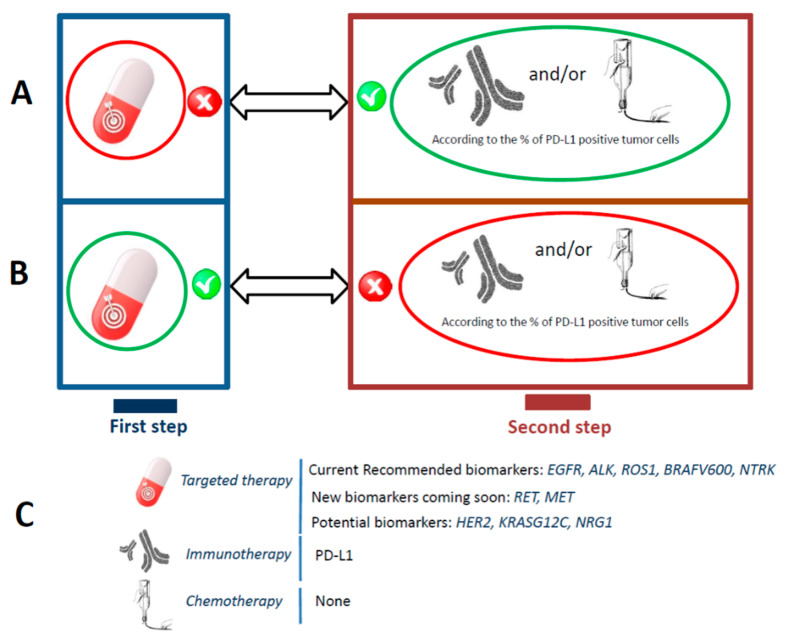
Main algorithms for the treatment of late stage non-epidermoid non-small lung cancer at diagnosis and strategies for biomarker testing. The first step concerns assessment of different genomic alterations among currently recommended genes. If the genes are wild-type (**A**) no targeted therapy is administered and patients receive immunotherapy alone (if PD-L1 is expressed in more than 50% of tumor cells) or in association with chemotherapy (if PD-L1 is expressed in less than 50% of tumor cells). If one active driver genomic alteration is detected on one of the currently recommended genes (**B**), no immunotherapy alone or in combination with chemotherapy should be administered since the patient should be treated with a targeted therapy. (**C**) List of the currently recommended and future molecular biomarkers for assessment at baseline.

Genomic studies on patients with lung cancer have led to the discovery of prognostic factors and predictive biomarkers of therapeutic agents targeting genetic alterations [1,2,3,4,5,6]. In recent years, advanced and metastatic non-small cell lung cancers (NSCLC) have benefited from an increase in the number of drugs for targeted therapy or immunotherapy, at diagnosis or on tumor progression [7,8,9,10,11,12,13,14,15]. Consequently, a progressive improvement in the overall survival of these patients has been observed [16]. Current therapeutic strategy in naïve-treated late stage NSCLC is based first on the presence of a genomic alteration actionable by a targeted therapy allowing to a personalized treatment (Figure 1A). Then, in the absence of a molecular actionable driver, the majority of patients received an immunotherapy alone or in association with a chemotherapy (Figure 1B). Different molecular targets are identified today and need to be assess, but others are coming soon and in the near future (Figure 1C). Due to the increase in the number of potential targets and genes for evaluation, sequential analyses for assessment of genomic alterations have been gradually replaced by next-generation sequencing (NGS) approaches [17,18,19,20,21,22,23]. NGS is a particularly attractive method since it evaluates in one step the mandatory molecular targets currently defined by international guidelines [24,25,26,27]. Currently, NGS analyses are mainly done at diagnosis with a tumor biopsy (TB), or, on tumor progression, with a TB and/or a liquid biopsy (LB). According to the algorithms defined by a clinical and molecular pathology laboratory and/or by the care organization, the NGS approach at diagnosis in late stage NSCLC can be used with a TB as a reflex method for *EGFR* (*Epidermal Growth Factor Receptor*)*, ALK (Anaplastic Lymphoma Kinase*)*, ROS1 (V-Ros Avian UR2 Sarcoma Virus Oncogene Homolog 1*), *NTRK* (*Neurotrophic Tyrosine Kinase*) and *BRAF* (*V-Raf Murine Sarcoma Viral Oncogene Homolog B1*) “wild-type” tumors (i.e, tumors with no genomic alterations on all these genes) with less than 50% of positive PD-L1 (Programmed Death-Ligand 1) tumor cells [28,29,30].

Recently, the use and analysis of extracted circulating free nucleic acids from plasma samples of a LB for NGS at diagnosis of late stage NSCLC emerged as a new concept, a complementary or even more an alternative approach to a TB NGS [31]. Many reasons drive the physicians to choose this direction: beside the well-known noninvasive advantage of a LB it is also repeatable, does not need hospitalization, and provides a more rapid result than TB NGS [31,32]. Moreover, the analyses performed with blood samples take into consideration the molecular heterogeneity of the tumor [33].

This review addresses the main published studies into the use of a LB for NGS at diagnosis of late stage NSCLC and deals with the advantages and limitations of this approach, notably for future development in a routine clinical practice.

## 2. NGS with Blood Samples at Diagnosis of Advanced Non-Small Cell Lung Carcinoma

**Figure 2:**
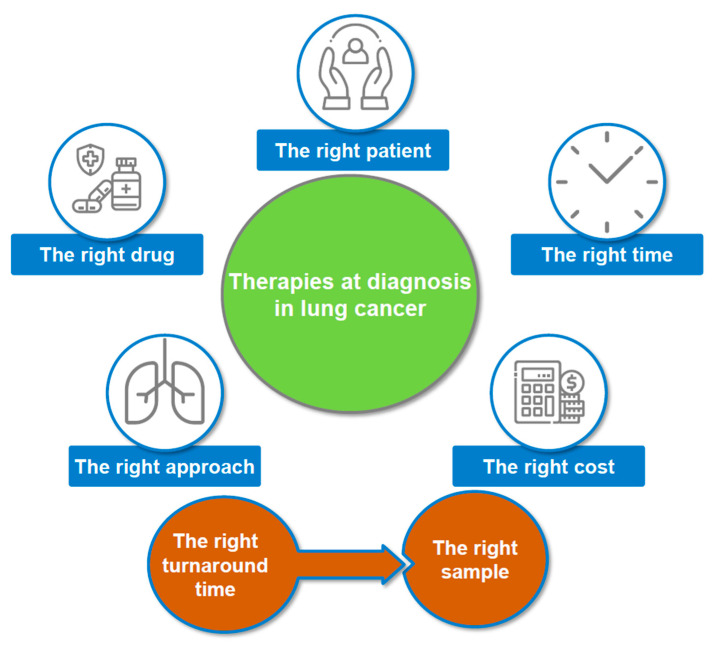
Different parameters to take into accounts at diagnosis in late stage non-small cell lung carcinoma to ensure optimal management and the best care of patients.

Therapies at baseline in advanced NSCLC rely on many different factors (Figure 2). A LB at diagnosis in late stage NSCLC was initially performed to detect genomic alterations in *EGFR* [20,21,34,35]. Notably activating *EGFR* mutations can be detected in LB from NSCLC, which is now performed in the daily practice of many clinical centers [36,37,38]. More rarely, *ALK* rearrangements can be assess in LB at diagnosis in these patients using a targeted sequencing approach [39,40,41]. However, only a few NGS studies using LB at diagnosis are currently available, despite the major interest of physicians in obtaining a rapid and broad evaluation of the genomic alterations in advanced NSCLC [42,43,44]. However, some recent prospective validation and feasibility studies performed at diagnosis in these patients showed good concordance between tissue- and plasma-based testing with NGS, giving consistent highly positive predictive values [42,43,44]. The NILE (Noninvasive versus Invasive Lung Evaluation) and the BFAST (Blood First Assay Screening Trial) studies are so far the most advanced studies in this domain [42,43]. The NILE study compared the sensitivity and the specificity of NGS analyses from matched LB and TB at diagnosis of 282 lung cancer patients from 28 institutions in the USA [43]. Moreover, the turnaround time (TAT) to obtain the results was evaluated. NGS from circulating free DNA (cf-DNA) was done using the Guardant 360 panel (Guardant Health, Inc., Redwood City, CA, US) [43]. The concordance between the results obtained from TB and LB approached 100% for *EGFR, ALK* and *BRAF* [43]. The study showed that of the 32% of patients who had targetable genomic alterations cf-DNA identified 27% [58% including *V-Ki-Ras2 Kirsten Rat Sarcoma 2 Viral Oncogene Homolog (KRAS)*] of the alterations, whereas tissue testing identified 21% of these alterations. Plasma testing yielded detectable tumor cf-DNA and complete profiling for 95% of patients. In contrast, tissue genotyping for all the eight National Comprehensive Cancer Network-recommended biomarkers was complete in only 18% of patients [43]. In this study, up to 20% of the patients did not obtain successful tissue testing for *EGFR* and *ALK*. However, it is noteworthy that the sample size of the *ALK* and *BRAF* cohorts of the positive patients for comparison of TB and LB in this study was very small, and an independent validation study is now certainly mandatory to confirm the results [43]. The results obtained with cf-DNA were reported in a median of nine days compared with 15 days for tissue testing, with a TAT of six days for cf-DNA by the end of the study. An initial plasma approach would have identified 87% of patients with actionable molecular alterations in the NILE study while an initial tissue approach would have identified only 67% of patients [43]. BFAST is an ongoing multi-center, open-label, multi-cohort study evaluating the relationship between blood-based NGS detection of actionable genetic alterations including *ALK* fusions and the activity of targeted therapies and immunotherapy in patients with treatment-naïve advanced NSCLC [42]. Of the 2219 patients screened by blood-based NGS, 119 patients (5.4%) had an *ALK* positive disease and 87 patients were enrolled and received alectinib [42]. *Echinoderm Microtubule-associated protein Like 4 (EML4*) was the fusion partner in 73 (84%) patients, with *Tumor Protein P53 (TP53)* mutations detected in 38 (44%) patients. Blood-based detection of *ALK* fusions brings clinical benefit to patients receiving alectinib [42]. Thus, a confirmed response rate of 87.4% and a 1-year progression free survival rate of 78.4% were reported, consistent with registration studies based on tissue profiling [42]. These data validated the clinical utility of blood-based NGS as an additional method to inform clinical decision-making for lung cancer patients with an *ALK* rearrangement [42].

A few other studies investigated NGS for detection of genomic alterations at diagnosis used blood from late stage NSCLC patients [44,45,46]. A study on a limited number of 21 NSCLC patients compared an in-house analysis with a limited panel of 11 genes (Oncomine, ThermoFisher Scientific, Waltham, MA, USA) and an outsource analysis with a panel of 70 genes (Foundation Medicine, Cambridge, MA, USA) [45]. This study showed a high level of concordance of detected genomic alterations in the common genes present in these two panels, but a shorter TAT to obtain the results when using the in-house approach [45]. A study was performed with a customized NGS panel (called SiRe) including six genes [*EGFR, KRAS, NRAS (NRAS Proto-Oncogene, GTPase), BRAF, KIT Proto-Oncogene Receptor Tyrosine Kinase (KIT)), Platelet-Derived Growth Factor Receptor Alpha (PDGFRA)*] for 194 patients with advanced adenocarcinomas [46]. A *KRAS* mutation was identified in 18.6% of patients [46]. It is noteworthy that many studies have been performed using targeted sequencing, notably digital polymerase chain reaction (PCR) and real-time (RT) PCR approaches, for the detection of *KRAS* mutations in cf-DNA [47,48,49,50]. The evidence that the *KRAS* genotype detected in cf-DNA may not reflect good prognosis of survival in NSCLC patients and the predictive role of this detection are controversial [47]. However, recent studies demonstrated that the presence of detectable *KRAS* mutation in plasma at diagnosis was associated with worse overall survival at stages I-IV of NSCLC [49,50]. Interestingly, since it is of strong interest to look more specifically for the *KRAS p.G12C* mutation for possible selection of metastatic NSCLC patients for AMGG510 or MTRX89 therapeutic strategies, one recent study showed the feasibility of detection of this mutation from cf-DNA with a high specificity and sensitivity at baseline [48]. In a study by Remon et al., the feasibility and effectiveness of an amplicon-based NGS assay (InVisionSeq, Inivata, Research Triangle Park, NC, USA and Cambridge, UK) with cf-DNA analysis for routine molecular profiling was assessed prospectively in daily practice for patients with advanced NSCLC to identify clinically relevant mutations and evaluate those for whom tissue sequencing could not be conducted or was not performed [44]. Ninety-four patients of the treatment-naïve cohort had successful and concurrent TB and LB molecular profiles. The sensitivity was 72% and increased to 81% for the defined core gene variant panel of gene hotspots within *EGFR, MET Proto-Oncogene, Receptor Tyrosine Kinase (MET), Erb-B2 Receptor Tyrosine Kinase 2 (ERBB2), BRAF, Serine/Threonine Kinase 11 (STK11)*, and *KRAS*. Overall, concordance for the broader panel in which concurrent tissue testing was performed was 95%, the sensitivity and specificity were 72% and 97%, respectively [44]. Finally, a cf-DNA profile for only 9% of patients was not obtained because of insufficient sequencing depth [44]. Several clinical trials assessed the interest of evaluating the blood-based tumor mutational burden (bTMB) at diagnosis as a predictive biomarker for response to immunotherapy [51,52,53]. The bTMB was calculated using NGS with cf-DNA and panels of different sizes [51,52,53]. It is noteworthy that different cut-off values (from 16 mut/meg to 20 mut/meg) were used for the different studies to define a high bTMB value predictive of an immunotherapy response [51,52,53].

## 3. Advantages of Using Liquid Biopsy Next-Generation Sequencing at Diagnosis

**Figure 3:**
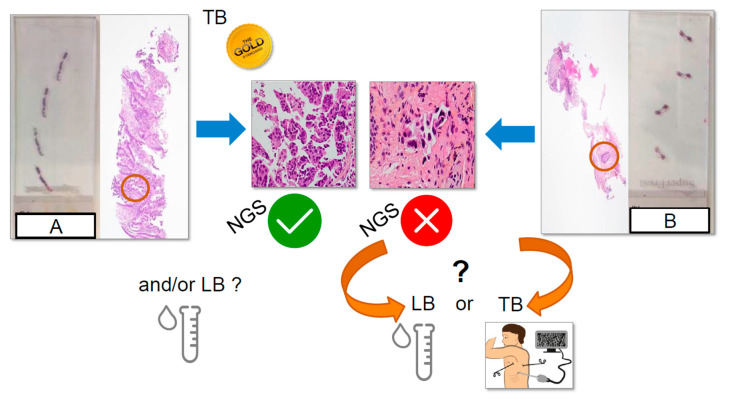
Different situations at diagnosis of late stage non-small cell lung carcinoma for the use of next-generation liquid biopsy. The tissue biopsy (TB) is still the gold standard for molecular analysis, but a liquid biopsy (LB) is a useful tool when facing the challenge of a low percentage of tumor cells in the TB. (**A**) a transthoracic biopsy of “high quality” with many tumor cells. Next-generation sequencing (NGS) from tissue can be done. However, in some patient, LB at diagnosis can also be discussed. (**B**) a bronchial biopsy of “poor quality” with a few tumor cells. NGS cannot be done. A renewed TB and/or a LB can be done to perform NGS.

Looking for molecular targets in treatment-naïve NSCLC is currently done systematically by TB, the gold standard approach for histological diagnosis, but also for PD-L1 status evaluation and for genomic analysis made from extracted somatic nucleic acids. However, this biopsy, notably when the lung tumor is peripheral, can be of very small size and/or can show a low percentage of tumor cells. Hence, the extracted nucleic acids obtained from this biopsy can be of poor quantity and/or quality for further genomic studies, notably when using an NGS approach (Figure 3). Using extracted circulating nucleic acids from plasma samples for NGS approach has several advantages (Table 1). Hence, a noninvasive and repeatable LB may be an option to detect molecular target(s) for first-line treatment in these patients. A NGS LB can be done initially at baseline, in a complementary manner or as an alternative to a TB. More often a NGS LB is done at diagnosis when it is the only option for the evaluation of genomic alterations.

So far, LB for lung cancer patients has been shown to be of strong interest during tumor progression, notably for tracking different mechanisms of resistance that can be targeted by different therapeutic agents [30,54,55,56,57,58]. Initially LB was orientated to targeted sequencing in *EGFR*, while looking for the *T790M* mutation in patients treated with first- or second-line tyrosine kinase inhibitors (TKIs) [20,21,37]. However, the mechanisms of resistance at tumor progression can be complex and can involve different mutations, amplifications or fusions in different genes, which limits the interest of using a targeted sequencing approach for tracking these mechanisms [55,59,60]. This is particularly the case for patients receiving a third-generation EGFR TKIs who may present many different genetic alterations on tumor progression [20,21]. So, using targeted sequencing for one gene with a LB is limiting and this highlights the strong interest of using a NGS method. Similarly, resistance mechanisms, notably *ALK* mutations can be investigated with a LB on tumor progression in patients treated with ALK inhibitors [55]. The presence of *ALK* mutations can be associated with some specific targeted therapies [41,55,61,62]. However, the sensitivity of NGS with a LB could be lower than that with a TB, notably for the detection of gene amplifications and fusions, which makes renewal of a TB of interest on tumor progression [63].

A LB at diagnosis in late stage lung cancer has been developed using mostly targeted sequencing, notably for the detection of *EGFR* mutations or *ALK/ROS1* fusions [21,37,40,55]. However, as a tumor progresses, NGS with a LB at baseline allows many genomic alterations on different genes to be detected and holds many advantages in comparison to NGS with a TB. Hence, a LB can easily replace a TB in case of a tumor site that is not accessible for biopsy or in the case of a fragile patient for whom a TB is more invasive. Similarly, in the case of a low quality/quantity of tissue and extracted nucleic acids, repeating a LB is definitively easier than doing a renewed biopsy during endoscopy or transthoracic puncture to obtain extracted nucleic acid for NGS analysis [64]. The TAT to obtain NGS results is in most situations faster with a LB than with a TB, which may allow more rapid administration of a targeted treatment in the case of a rapid progression of a tumor [43,65]. The TAT for NGS results from a TB can be much longer than for a LB depending on the clinical organization, the workflow of the samples and the pre-analytical, analytical and post analytical steps [31,43]. The international guidelines currently require the *EGFR, BRAF, ALK, ROS1, NTRK* status to be obtained within less than 10 days, and for some experts within 5 days [12,65]. However, we assume that these TAT cannot be reached by many organizations when using NGS with a TB. Molecular biology analyses using a LB can avoid hospitalization for a bronchial endoscopy or transthoracic biopsy and thus can also be more cost effective [66]. Analysis of circulating plasma free DNA can reflect the molecular status of different tumor sites (primary tumor and one or several metastases) and can allow better detection of some targeted genomic alterations that may not be visible on a TB due to the tumor heterogeneity [59,67,68,69,70]. One of the issues that can be eliminated using a LB for NGS, notably when taking blood on EDTA tubes, is the appearance of artefacts, notably DNA deamination due to the formalin fixative, which is the main fixative used for TB [71,72].

The tumor mutational burden (TMB) can be evaluated from a TB but also from a LB. One advantage of the evaluation of a bTMB is that the TMB heterogeneity is taken into consideration, which can be evaluated for different tumor sites [73,74]. TMB assessment can vary according to the size of the gene panel, the sequencing technology, but also to the different pre-analytical conditions, notably the time for formalin fixation [72,75,76,77]. Evaluation of the TMB with blood has been done at diagnosis for NSCLC and used as a predictive biomarker of immunotherapy [52,75]. However, one of the drawbacks is the definition of the cut-off of a high bTMB. A high bTMB is variably defined according to the clinical trial, the therapeutic strategy, and the panel of genes used [52,75]. So, different international initiatives aimed at harmonizing the results of TMB obtained with different panels of genes have been developed [78,79].

Finally, one of the major interests in using a LB for a NGS approach at diagnosis is to increase the number of patients included into clinical trials, since this noninvasive approach can allow better and faster selection of patients who may benefit from newly developed therapeutic molecules [80,81,82].

## 4. Limitations and Drawbacks of NGS with a Liquid Biopsy for Naïve-Treated Advanced Non-Small Cell Lung Carcinomas

Looking for genomic alterations with LB NGS at diagnosis in advanced lung cancer holds a few limitations, notably in comparison to TB NGS (Table 2). Different studies demonstrated discrepancies between the results of NGS from matched LB and TB obtained from the same patient [83,84,85,86,87]. These discrepancies can be explained by biological and/or technical issues. First, the quantity of cf-DNA can vary according to the histological subtype and according to the tumor stage [88]. Some patients with stage IIIB/IV, notably with oligometastic disease, have no detectable or a low amount of ct-DNA. Additionally, it is well-recognized that certain tumors from a few metastatic sites (notably the brain) do not shed or shed very little tumor DNA into the blood stream [89]. Certain tumors progress very slowly and have a low index of proliferation and thus a low amount of cf-DNA [90]. Moreover, some tumors with specific mutations in certain genes (such as *KRAS* and *P53*) are associated with a higher level of cf-DNA while other tumors with some mutations, such as *EGFR* mutations, are frequently associated with a lower level of cf-DNA [90]. Certain types of genomic alterations can be more difficult to identify with circulating nucleic acids than with tumor tissue. Notably, some gene amplifications (such as *MET, RET* and *ALK* amplifications) or rearrangements [such as *ALK, ROS1, Proto-Oncogene Tyrosine-Protein Kinase Receptor Ret (RET), NTRK,* and *Neuregulin 1 (NRG1)* rearrangements] are less frequently detectable in a LB that in a TB. However, some sequencing technologies need a higher amount of nucleic acid than others and some of these technologies may also detect, with a higher sensitivity, gene amplifications and rearrangements.

At baseline, the diagnosis of a lung cancer cannot be done using LB NGS, since a TB is the gold standard for histological characterization. Moreover, even if a LB can give information concerning the assessment of the PD-L1 status obtained from PD-L1 expression analyses of plasma and/or circulating tumor cells (CTCs), it is mandatory to evaluate the PD-L1 status on only cytological and/or tissue samples [73,91,92].

Some pitfalls can result from the presence of clonal hematopoiesis, which could associate the presence of some mutations (notably *KRAS* mutations) on circulating free germinal DNA, notably for the elderly [93,94,95,96,97]. So, the analysis of different variants needs a high level of bioinformatic expertise to distinguish these different germinal mutations from somatic mutations. Finally, different mutations can be present in circulating germline DNA (such as some *EGFR* mutations) and have to be distinguished from somatic mutations [98,99].

Gene panels used for NGS in clinical care are different in size and composition. They can contain a very low number of genes or up to at least 500 genes [87,100,101,102,103,104]. So, different panels of genes are used for LB NGS [105]. These panels include a variable number of genes of interest. According to the sequencing technique some panels need more or less nucleic acid. Therefore, the different NGS approaches can give a variable sensitivity and specificity [35,106,107,108,109]. So according to the technology used (amplicon based or hybrid capture based sequencing) the quantity of nucleic acid needed for a NGS analysis with a LB has to be discussed according to the indication, i.e., looking for a limited or a large number of genes, and/or the importance of assessing the bTMB. A recent study showed that correlation of the different TMB values evaluated with different panels and technologies was quite low when assessed with cut-off values from 5 to 25 mutations per megabase [77]. It should be highlighted that these cut-off values are mainly used in most of the clinical trials, notably in thoracic oncology in the domain of immuno-oncology. For this, no comparative studies have so far been set up with blood samples for the assessment of the different TMB panels. The different cut-off values of the bTMB vary a lot according to the panels and the therapeutic molecule. Additionally, to compare the TMB values from the primary tumor site or different metastatic sites with those of the bTMB seems to be an issue, since the latter should correspond to the addition or the average of the different TMB values existing at the different tumor sites. Finally, the different buffers used for blood sample management may contain a low amount of formalin, which induces deamination. The discrepancies between most of the panels used for the assessment of tissue and bTMB highlight the difficulties in obtaining harmonious studies for bTMB evaluation.

It is certainly more difficult to obtain accreditation for NGS with a LB than with a TB due to the fact that the validation of multiple genetic alterations detected with the cf-DNA requires enough material to be obtained for transfer to the different laboratories for external control in comparative studies. Moreover, the validation by the Food and Drug Administration of some companion diagnostic tests should be more difficult when using an NGS approach with LB than with TB [110].

## 5. NGS with Blood at Diagnosis of Advanced Non-Small Cell Lung Carcinoma: How to Optimize?

Due to some limitations of making a LB for NGS as described above, a couple of actions could improve the use of blood samples from lung cancer patients for NGS development (Table 3).

### 5.1. Improving the Quality and Quantity of Circulating Nucleic Acid

One of the major current challenges in the domain of LB concerns increasing the quantity and quality of the extracted nucleic acid so as to use large gene panels and avoid false negative and/or false positive results [111]. However, the following question could be asked: are we able to increase the level of extraction of nucleic acids from plasma with new technologies and/or new improvements in the pre-analytical phases? This is of significant importance when using a LB for NGS at diagnosis since according to the tumor and/or to the different metastatic sites, the quantity of cf-DNA at baseline may not be sufficient in quantity for robust analysis of the different genomic alterations [112]. In this context, different options can be considered: (i) increasing the volume of the blood sample taken from the patient to obtain a higher quantity of nucleic acid after extraction. However, it does not seem possible to get more than 20 mL from patients with metastatic lung cancer (the average volume is 10 mL of blood in daily practice); (ii) optimize the pre-analytical steps by using an efficient buffer that limits degradation of leucocytes and thus the release of germinal DNA from these cells into the blood; (iii) reduce as much as possible the time between blood puncture and the centrifugation steps and (iv) use some new reagents that increase nucleic acid extraction from plasma and thus optimize the ratio between the available plasmatic volume and the amount of extracted nucleic acid [111,112,113,114,115,116,117,118,119,120,121].

The guidelines allowing optimization of the procedures of the different analyzes using LB have to be better standardized. Multi-centric and independent validation studies need to be systematically set up to evaluate the reproducibility and the robustness of the different techniques as well as to better control the different steps of the pre-analytical phases [122,123,124]. So, a number of initiatives aimed at establishing new recommendations and guidelines have started to emerge [125,126,127].

### 5.2. Assessment of the Genomic Data

Orthogonal methods need to be set up regarding some discrepancies between the different approaches and the different panels used for NGS assessment [128]. Discrepancies between the results of NGS obtained from matched tissue and blood samples could be explained by the tumor biology, the different sensitivities of the technical approaches and the fact that different panels were used for the different studies, which can lead to variable sensitivities and specificities [38,104,128,129]. Moreover, the demonstration of incidental germline mutations is possible according to the different genes and mutations detectable with NGS. This leads to the question of who is going to validate these results and give the results to the physicians and patients depending on the discovery of some constitutional genetic mutations [130].

### 5.3. Integration of Different Components of Interest in Blood

**Figure 4:**
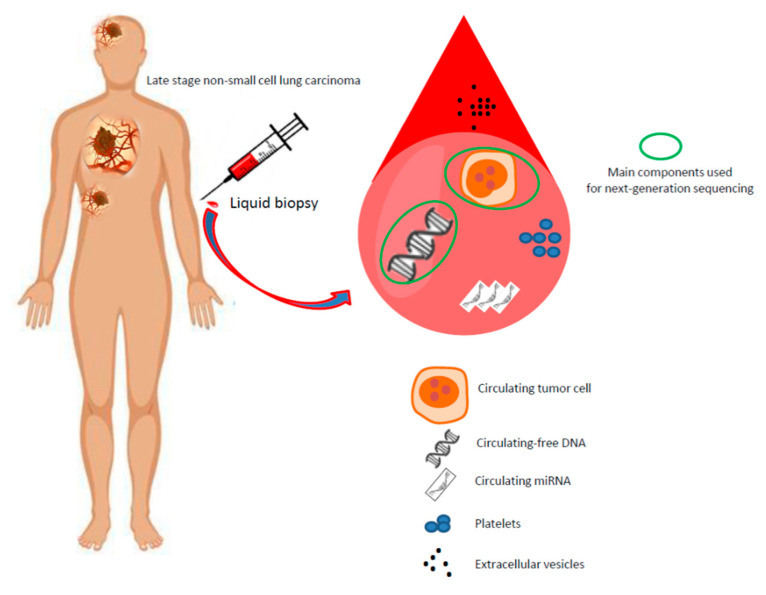
Circulating blood components in patients with late stage non-small cell lung carcinoma. Several components can be isolated at diagnosis (baseline), including circulating free-DNA, circulating tumor cells, microRNA, platelets, and extracellular vesicles (mainly exosomes) for biomarker assessment. The majority of next-generation sequencing technologies are currently being developed from circulating free-DNA and from nucleic acids extracted from circulating tumor cells (green circles).

Many clinical trials performed on advanced NSCLC patients at baseline are based on analyses made with cf-DNA. However, other blood components of patients such as CTCs, extracellular vesicles (EVs), including exosomes, platelets and microRNAs are of interest [131,132,133,134,135,136,137,138,139,140,141,142] (Figure 4). The integration of analyses of different components may optimize in the near future the global biological information necessary to make better strategic therapeutic decisions [137,143]. However, sequencing a CTC genome or studying the transcriptome are associated with technical issues [144,145,146]. So, obtaining a sufficient number of CTCs for library preparation and NGS is certainly the main critical step in CTC sequencing. Moreover, some tumors tend to shed more cells in the blood stream than other tumors, even independently of the tumor stage, so the number of CTCs can vary from zero to a hundred and even thousands per 7.5 mL of blood. Indeed, obtaining enough CTCs for sequencing and NGS still remains an important issue for NSCLC, which limits the current number of CTC sequencing studies available in the literature. CTCs loss and/or DNA damage during enrichment, isolation, and/or genome amplification can have a high impact on the quality of the results [144,145,146]. Accumulating evidence has revealed that EVs, notably key exosomal cargo, are significantly mis-regulated in tumors and can serve as diagnostic, prognostic, and predictive biomarkers for lung cancer [131,134,138]. Moreover, the issue as to which isolation method of EVs to use for a given downstream application such a NGS is currently controversial and as yet to be settled, notably for the use of this approach in routine clinical practice [131,134,138,147,148,149]. CTCs can activate and educate platelets [140]. Indeed, platelets can ingest mRNA from cancer cells, triggering a possible modification in the platelet transcriptome that reflect the tumor profile. So, platelets are considered important repositories of potential RNA biomarkers (mRNA, miRNAs, circRNA, lncRNA, and mitochondrial RNA), including biomarkers for NSCLC detection [150]. During the last decade, a new promising group of biomarkers has appeared and its use for cancer diagnosis and monitoring is being intensively studied—the miRNAs [136,139,142]. Currently, circulating miRNAs are promising markers for lung cancer diagnosis, prognosis, monitoring the treatment response, and as powerful tools for personalized approaches [136,139,142]. Taken together, these novel programs raise some technological but also cost-effective challenges as well as those associated to complex data analyses, which could be obtained by the combination of results [151].

## 6. Conclusions

Recent reviews have highlighted the great opportunity that represents the use of a LB as a tool for diagnosis, prognosis and/or the discovery of predictive biomarkers in oncology, notably by developing associated NGS tools [152,153,154,155]. This underlines the importance of rapidly setting up this approach in the daily clinical practice for improvement to care of lung cancer patients. Currently, LB NGS on tumor progression is beginning to be established in some comprehensive cancer centers but is not adopted as much at diagnosis and is still under heated discussion, notably for its usefulness in comparison to some targeted sequencing tests [21,156,157]. Moreover, there is still a gap between the use of LB and TB NGS at baseline in advanced NSCLC, underlying the fact that TB is still currently the best approach and the gold standard for diagnosis and detection of genetic alterations in these patients [158,159].

A LB for detection at diagnosis of some molecular therapeutic targets in advanced NSCLC, or even in squamous cell lung cancers, is a very promising new approach for the oncologist, thus avoiding performing an invasive tissue biopsy [73,160,161]. The importance of this concept is highlighted by recent technological developments for LB NGS at baseline and the setting up of different clinical trials. However, even if this is a very exciting new area it is important to be aware of the message the physicians give to their patients concerning the current limitations of NGS performed with circulating nucleic acids at diagnosis, knowing that negative results could be due to the lower level of performance of molecular biology analyses with blood compared with TB. Hence, false negative and even more false positive results with LB NGS can be detrimental to choosing the right therapeutic strategy for patients [96]. Currently it seems that a TB at diagnosis is mandatory in advanced NSCLC, since some biomarkers such as PD-L1 need to be performed only on tumor tissue sections using immunohistochemistry. Moreover, it is much more efficient to look for gene amplifications or rearrangements with a TB than with a LB, despite some recent results showing good concordances. However, the latter results need to be confirmed outside of clinical trials, notably in routine clinical practice [162,163,164,165,166,167]. Looking for *MET* and other gene amplifications or for gene fusions (on *ALK, ROS1, RET, NTRK, NRG1*) at diagnosis with a LB seems to be hazardous without complementary research into TB [158,159,160]. The TB is still the gold standard approach for most physicians at diagnosis [84,158,159,168,169,170]. However, a combined approach associating at the same time an NGS analysis on matched TB and LB could be of strong interest in aiming to establish a complete molecular portrait of the tumor, which can take into consideration the genomic alteration of the primary and the metastatic site(s) [171,172,173,174,175]. Though rare, if no tissue is available at diagnosis LB NGS is the only alternative in identifying a molecular alteration accessible to a targeted therapy [170,176,177,178]. LB NGS can also be a means to speed up the care of lung cancer patients in certain situations, which do not allow molecular results to be obtained from a TB in an acceptable TAT for administration of an appropriate treatment [66].

Finally, different studies have examined the perspectives of using LB for early cancer diagnosis [179,180]. However, in a daily practice the majority of the studies did not lead to its use in routine practice by physicians [176,177,178,179,180]. One perspective of LB NGS concerns detection of lung cancer at an early stage or even for the prediction of lung cancer onset in a population at high risk, such as heavy smokers with chronic obstructive pulmonary disease [179,180,181,182,183]. Currently these approaches, even if of great interest, are not available in most of the clinical centers for care. The major limitation is the low level of shedding of cancer cells into the blood in this population of patients [35,112].

## Figures and Tables

**Table 1:** Advantages of using next-generation sequencing (NGS) with circulating free (cf) nucleic acids extracted from blood samples at diagnosis of non-small cell lung carcinoma.

Screening of many genomic alterations on several genes at the same time with a noninvasive, painless and repeatable approach
Can be done in a complementary manner or as an alternative to a tissue biopsy
Can be the only option for genomic alteration assessment in certain patients with no possibility of doing a tissue biopsy
Can be the only option for genomic alteration assessment in the case of a low quality and/or quantity of extracted nucleic acid from a tissue sample
The turnaround time (TAT) for NGS results with cf-nucleic acid is faster than for NGS from nucleic acid extracted from a tissue biopsy
NGS of blood samples is globally and indirectly cost effective compared to NGS from a tissue biopsy since avoiding patient hospitalization
NGS of blood samples can reflect the molecular status of different tumor sites at the same time
NGS of blood samples taken with EDTA buffer tubes can avoid artifacts associated with DNA deamination due to the effect of the formalin fixative
Evaluation of the tumor mutation burden (TMB) using NGS with blood samples can integrate the TMB heterogeneity from different tumor sites and at the same time
NGS with a liquid biopsy can allow an increase in the number of patients included into clinical trials at diagnosis

**Table 2:** Limitations of using next-generation sequencing (NGS) with circulating free (cf) nucleic acids extracted from blood samples at diagnosis of non-small cell lung carcinoma.

The quantity of cf-nucleic acid extracted from plasma samples may not be sufficient for NGS due to the tumor stage
Brain metastases usually shed a too low amount of tumor cf-nucleic acid into the blood for NGS
Some specific mutations in certain genes are associated with a low amount of cf-nucleic acid for NGS
Gene amplifications and rearrangements are less frequently detectable with cf-nucleic acid in blood samples as the same nucleic acid extracted from a tissue biopsy
Assessment of PD-L1 for first-line immune check point inhibitor treatment is not possible with blood samples
Pitfalls can be associated to NGS with cf-nucleic acid due to clonal hematopoiesis on circulating free germinal DNA
Validation and accreditation processes are more difficult to set up for NGS with blood samples than for NGS with a tissue biopsy

**Table 3:** Opportunities for improvement of next-generation sequencing (NGS) with blood samples containing circulating free (cf)—nucleic acids at diagnosis of non-small cell lung carcinoma.

Optimize the pre-analytical steps using new buffers that limit the degradation of circulating blood hematological cells
Reduce the time between veinule puncture and centrifugation of the blood
Develop new procedures and reagents to increase the amount of nucleic acid extracted from plasma
Increase the number of multicenter studies that compare the different gene panels used for NGS with cf-nucleic acid
Integrate NGS with cf-nucleic acid and from other blood components such as circulating tumor cells and/or circulating extracellular vesicles
Reduce the volume of the samples of plasma for NGS analyses with cf-nucleic acid for routine clinical practice

## Data Availability

Not applicable.
